# Charting electronic-state manifolds across molecules with multi-state learning and gap-driven dynamics via efficient and robust active learning

**DOI:** 10.1038/s41524-025-01636-z

**Published:** 2025-05-13

**Authors:** Mikołaj Martyka, Lina Zhang, Fuchun Ge, Yi-Fan Hou, Joanna Jankowska, Mario Barbatti, Pavlo O. Dral

**Affiliations:** 1 University of Warsaw, Faculty of Chemistry, 02-093 Warsaw, Poland; 2 State Key Laboratory of Physical Chemistry of Solid Surfaces, Department of Chemistry, College of Chemistry and Chemical Engineering, and Fujian Provincial Key Laboratory of Theoretical and Computational Chemistry, Xiamen University, Xiamen 361005, Fujian China; 3 Aix Marseille University, CNRS, ICR, Marseille, France; 4 Institut Universitaire de France, Paris, France; 5 Institute of Physics, Faculty of Physics, Astronomy, and Informatics, Nicolaus Copernicus University in Toruń, Toruń, Poland; 6 Aitomistic, Shenzhen 518000, China

**Keywords:** Theoretical chemistry, Light harvesting

## Abstract

We present a robust protocol for affordable learning of electronic states to accelerate photophysical and photochemical molecular simulations. The protocol solves several issues precluding the widespread use of machine learning (ML) in excited-state simulations. We introduce a novel physics-informed multi-state ML model that can learn an arbitrary number of excited states across molecules, with accuracy better or similar to the accuracy of learning ground-state energies, where information on excited-state energies improves the quality of ground-state predictions. We also present gap-driven dynamics for accelerated sampling of the small-gap regions, which proves crucial for stable surface-hopping dynamics. Together, multi-state learning and gap-driven dynamics enable efficient active learning, furnishing robust models for surface-hopping simulations and helping to uncover long-time-scale oscillations in *cis*-azobenzene photoisomerization. Our active-learning protocol includes sampling based on physics-informed uncertainty quantification, ensuring the quality of each adiabatic surface, low error in energy gaps, and precise calculation of the hopping probability.

## Introduction

Electronic-structure methods offer unique insight into complex photophysical and photochemical problems, helping to guide and rationalize the experimental results. Unfortunately, these methods come with a steep computational cost, which severely limits their practical applications, particularly in nonadiabatic molecular dynamics simulations^1^. The latter provides an invaluable computational tool for investigating complex photoprocesses in the real-time domain, yet requires performing a large number of expensive excited-state calculations, thereby constraining their applicability. Today, by far the most popular nonadiabatic dynamics simulation technique is trajectory surface hopping (TSH). It has been successfully used to study a wide range of photoresponsive systems^2–6^. TSH simulates the excited-state dynamics of molecules by propagating a swarm of independent classical nuclear trajectories on quantum electronic potential energy surfaces (PESs). Nonadiabatic events are included through interstate instantaneous hoppings, whose probability is evaluated at each integration time step on the basis of the coupling strength between the starting and the target PES. The swarm of surface hopping trajectories is expected to approximate the quantum nuclear wavepacket. TSH does not need global knowledge of the PESs, only of their values at the classical nuclear geometry. Thus, it is perfectly suited for on-the-fly simulations, in which electronic structure calculations delivering energies, energy gradients, and nonadiabatic couplings are executed as required in the course of the trajectory propagation. The main bottleneck of TSH simulations is their high computational cost due to the need to perform hundreds of thousands or even millions of single-point electronic-structure calculations. Hence, significant effort has been put into developing machine learning (ML) protocols to accelerate the TSH simulations of photoprocesses^7–24^.

As of today, cutting-edge ML models allow breaking through the limitations of the electronic-structure TSH simulations by enabling large-scale computations for longer times and with more quantum-classical trajectories. This research helped to uncover interesting photochemical phenomena, some of which were rather rare to be confidently quantified, or even detected, with the pure electronic-structure calculations^14,25–29^. Despite all this progress, the state-of-the-art ML-accelerated TSH remains an extremely computationally expensive undertaking, with no clear protocols, requiring intensive human-expert supervision. For these reasons, it is still often easier to perform non-ML, pure electronic-structure TSH. This state of affairs precludes the widespread adoption of ML-TSH by the community, which is reflected by the fact that only a few expert groups reported new photochemical phenomena based on ML-accelerated TSH and the fraction of publications using ML in TSH studies remains relatively small compared to the bulk of TSH simulations (circa 3%, as estimated comparing all Web of Science records on “Trajectory Surface hopping” to the number of records that also mention machine learning).

**Fig. 1:**
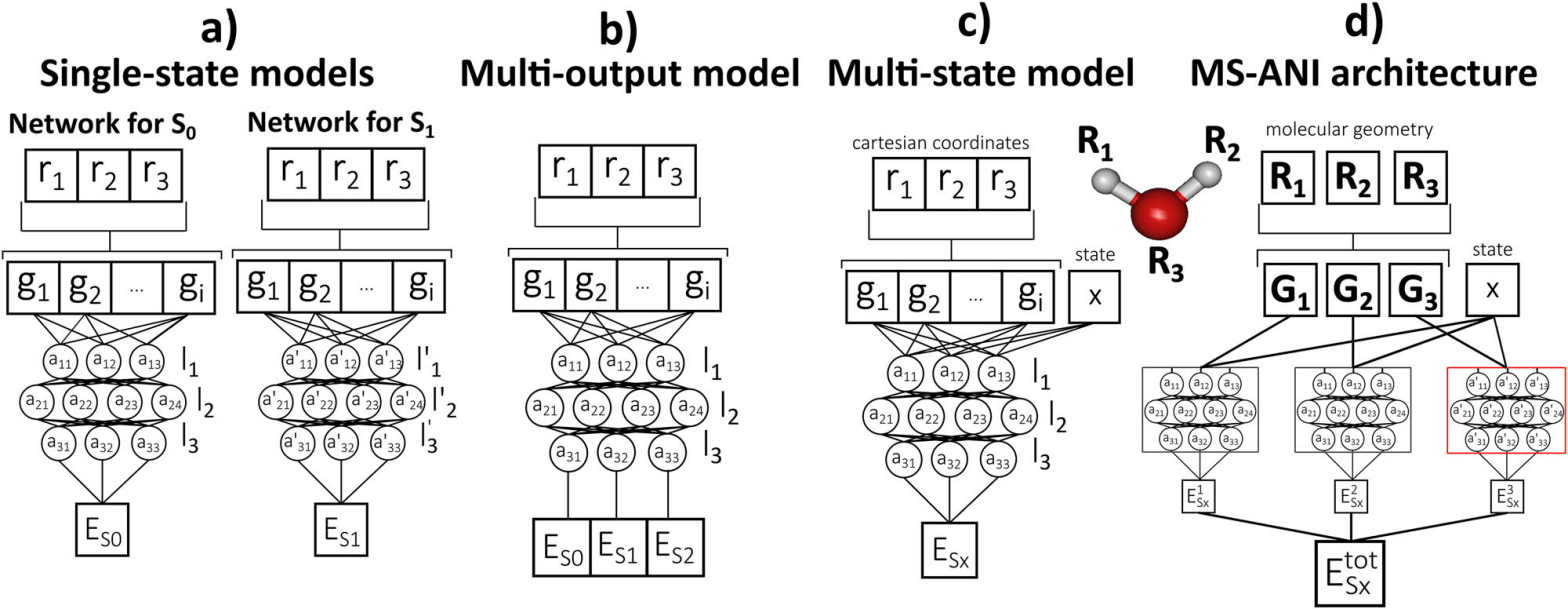
A comparison of different NN models that can be used for excited state properties prediction, using a triatomic molecule with coordinates R_*i*_ = {*r*_1_, *r*_2_, *r*_3_}. **a**–**c** show atomistic NN, highlighting the differences between the approaches used to predict excited-state energies: **a** shows a combination of single-output NNs; **b** – multi-output NN; **c** – multi-state NN. The vectors **G**_*i*_ = {*g*_1_, *g*_2_, . . . , *g*_*i*_} are the molecular descriptors of a given atom, *l*_*i*_ are the network layers with nodes *a*_*ij*_, and *E*_*Sx*_ is the predicted energy contribution of an atom to adiabatic state x. **d** shows the architecture of the MS-ANI model, which consists of separate NNs for each atom type, with all atom-wise predictions summed up to yield the total energy of the molecule for adiabatic state x, ${E}_{Sx}^{tot}$.

The fundamental issue undermining the advance of ML-TSH is the challenge of predicting a dense manifold of potential energy surfaces, with their complex topography and small interstate energy gaps. Learning this manifold, along with all the intrinsic correlations, with high precision is required for robust MLTSH. The most popular solutions suggested so far include creating single-state ML models (i.e., one ML model per each electronic state, Fig. 1a), as for example done by Westermayr et al. for the methylenimmonium cation^11^, or Hu et al. for 6-aminopyrimidine^8^, and multi-output ML models (i.e., a single neural network (NN) with the last layer containing as many output neurons as there are electronic states of interest), such as SchNarc^13^, SpaiNN^24^, and PyRAI^2^MD^14^. Both these solutions, unfortunately, have significant disadvantages: the single-state models do not capture correlations between the states, often leading to inferior performance in ML-TSH, while the multi-output models frequently suffer from more significant errors compared to single-state models, as shown later in this work. Learning excited-state PESs across different molecules is another big challenge, and only a few studies^16,30^ have attempted to do so until today.

To address these challenges, though, having a good ML model architecture alone is not sufficient: one must precisely map the topography of the electronic-state manifold in the regions visited during TSH. This is an arduous task. Some proposed solutions were based on extensive manual construction of the data sets and on generating data with the pure electronic-structure TSH dynamics^8,10,31^. At the same time, for the sake of universality and efficiency, it is desirable to build data sets from scratch using active learning (AL). However, the reported AL strategies based on ML-TSH exploration required manual adjustment of sampling criteria^11,14,26^. Even then, these strategies were often insufficient for robust ML-TSH and require further interventions, such as interpolation between critical PES points (e.g., between minima and conical intersections)^26,27,32^. Another critical issue preventing efficient ML-assisted NAMD is the need for easy-to-use, end-to-end protocols and software enabling routine simulations to be run by non-ML experts. However, progress is being made in this direction too^13,14,33^.

In this work, we solve these daunting problems by i) developing accurate and extendable physics-informed multi-state model capable of learning across chemical space (Fig. 1c), ii) proposing accelerated sampling of the critical small-gap regions in the electronic-state manifolds with gap-driven dynamics, and iii) implementing end-to-end, efficient, and robust active learning protocol based on physics and automatically, statistically determined criteria. We show that these new developments make the acceleration of TSH with ML affordable, as for not-so-flexible systems we can obtain the final simulation results within days on commodity hardware. The protocol also works for photoreactions albeit with more computational effort needed. Remarkably, our methods also enable learning an arbitrary number of electronic states not just for a single molecule but also simultaneously across different molecules and different reference electronic-structure levels.

## Results

### Multi-state learning

Here we introduce a novel ML architecture that: 1) can learn an arbitrary number of electronic states on equal footing with high accuracy, with the number of electronic states used independent of the NN structure 2) can make predictions for different molecules, 3) captures the required correlations between states and, especially, correctly reproducing energy gaps between surfaces, and 4) is easy (relatively fast) to train and evaluate. The core idea of this new implementation is captured in Fig. 1c.

The distinguishable feature of our model is the inclusion of information about the state ordering number (e.g., 0 for the ground state, 1 for the first excited state, etc.) in the model-processed features alongside the geometric descriptors. This leads to multiple benefits compared to either single-state or multi-output models reported in the literature previously (cf. Fig. 1a, b). The paramount benefit is that this architecture can handle an arbitrary number of states on equal footing. The information about all the states is passed through all hidden and output neurons of the NN, and these neurons differentiate between different states. This allows not only for electronic state information to be propagated through the entire network in the training process but also training on data with diverse labels (molecules with different numbers of labeled electronic states) and transfer learning between very different datasets. In contrast, in the single-state models, only information about one state is learned at a time, i.e., the correlation between states is essentially lost, which might lead to the inferior performance in ML-TSH, as was observed earlier^34^. In the multi-output models, on the other hand, only the last output layer differentiates between states. Still, the correlation between states is implicitly learned to some extent as the weights are shared in the preceding layers^35^. Eventually, it may lead to better performance in ML-TSH, despite the larger errors in energies.

Furthermore, the proposed multi-state model is ideally suited for capturing crucial photophysical information, such as the importance of energy gaps between states. Crucially, we found that the accurate treatment of small-gap regions is the key to the robust performance of ML models in TSH. As one of the solutions to the small-gap problem, we include the special loss term *L*_gap_ taking into account the error in the gaps, which ensures accurate prediction of energy gaps when training the multi-state models:

1 $${L}_{{\rm{gap}}}=| | \Delta {E}^{{\rm{ML}}}-\Delta {E}^{{\rm{ref}}}| {| }^{2},$$

where Δ*E* denotes the energy gaps between adjacent adiabatic states, and the superscripts ‘ML’ and ‘ref’ correspond to the NN prediction and reference values, respectively.

We include this loss in the total loss, *L*, containing the terms for energy, *L*_*E*_, and force errors, *L*_*F*_:

2 $$L={\omega }_{E}{L}_{E}+{\omega }_{F}{L}_{F}+{\omega }_{{\rm{gap}}}{L}_{{\rm{gap}}}.$$

The energy and force losses follow their usual definitions of ∣∣*E*^ML^ − *E*^ref^∣∣^2^ and ∣∣**F**^ML^ − **F**^ref^∣∣^2^, respectively. Each of the loss terms comes with the corresponding weight, *ω*, with *ω*_*E*_ and *ω*_gap_ equal to 1, treating the gaps and energies as equally important, and with *ω*_*F*_ set to the standard value of 0.1. This makes our multi-state model a representative of the physics-informed NNs, which were shown to provide more qualitatively accurate behavior in the related quantum dynamics context^35^.

The overall NN architecture is based on the established ANI-type network, which has a good balance between cost and accuracy^36^, and which we later compare with the more accurate yet slower equivariant networks. It means that we use the same ANI-type structural descriptors and have separate networks for each element type. The predictions are made for each atom separately and then summed up to yield the total energy for the requested state. The self-atomic energies are computed once and shared for all states of the model. We refer to this architecture as multi-state ANI (MS-ANI).

**Fig. 2:**
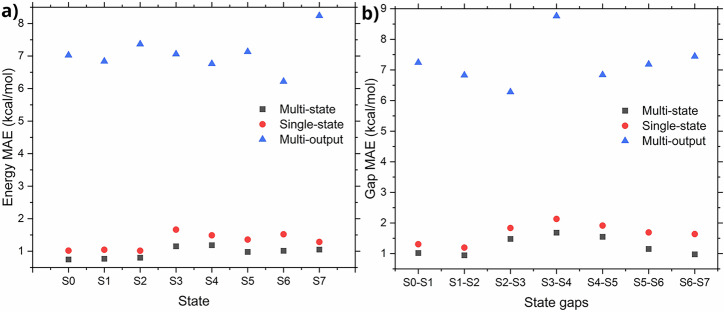
Performance of the different NN architectures. Multi-state (black squares), single-state (red dots), and multi-output (blue triangles) models are compared by predicting the energies (**a**) and energy gaps (**b**) of the first 8 electronic states of pyrene, and judged by mean absolute errors (MAEs).

To demonstrate the performance of the multi-state model, we first evaluate its accuracy in predicting the energies and energy gaps for the first eight electronic states of pyrene. To this end, we constructed a data set of 4000 molecular conformations generated from the Wigner sampling around the S_0_ minimum structure. All calculations were performed with AIQM1^37^ using CIS^38^ treatment for the excited-state properties. Eventually, we trained the multi-state model on 3000 conformations and evaluated it on the remaining 1000 test conformations. Remarkably, the model yielded mean absolute errors (MAEs) of energies close to ca. 1 kcal/mol (0.04 eV) (Fig. 2a) and root-mean-squared errors (RMSEs) below 1.6 kcal/mol (Table S1 of the ESI). The energy gaps were also described with good accuracy, with MAEs below 1.7 kcal/mol (Fig. 2b), and with the maximum root-mean-squared error (RMSE) below 2.2 kcal/mol (Table S2). Altogether, errors for energies and gaps are falling close to the chemical accuracy margin of 1 kcal/mol.

These calculations are better put in perspective when compared to the single-state and multi-output ANI-type models. One can observe that, in the multi-output model, for all electronic states, the errors in energies and gaps are substantially higher than in the multi-state and single-state models, with MAEs between 6.2 and 8.3 kcal/mol for energies, and 6.3 and 8.5 kcal/mol for energy gaps. When comparing the multi-state model with the single state model, we can see that MS-ANI outperforms it for all electronic states energies and gaps, with the differences being most pronounced for higher excited states and strongly coupled S3–S4 states (Fig. 2, as well as Tables S1–S3). In all of the single-state models’ MAEs and RMSEs are above 1 kcal/mol.

In addition to the multi-state model’s superior performance, its training required much less time than training the eight separate single-state models (45 minutes vs 3 hours on an RTX 4090 GPU).

We also observe, for the first time, that the accuracy of energies of the ground state is improved when information regarding the other, i.e., excited states, is included. Moreover, our multi-state model can learn the excited-states properties with the same or higher accuracy than a single-state model can learn the ground state: a previously unachievable result.

### Mapping small-gap region with gap-driven dynamics

Regardless of how good the model is, a sufficient amount of data to sample all relevant regions of the PESs for the TSH is always a necessity. This data is usually generated based on the TSH trajectories, either propagated with a reference electronic-structure method or with ML in an active learning loop. However, such data may have insufficient representation of the critical regions in the vicinity of the conical intersections because the TSH trajectories typically contain only very few points in that region representing a small fraction of all time steps in the trajectories. It is also known that ML models, in general, struggle with reproducing the PES near conical intersections^39^, hence extra steps must be taken to ensure the correct learning of low-energy-gap regions. This is particularly true for *S*_0_/*S*_1_ conical intersections, usually associated with more drastic geometric changes than excited-state crossings. This explains why some of the previous studies, even after training on extensive data sets (e.g., from the reference TSH trajectories), still could not obtain robust dynamics and needed to switch from ML to the reference electronic-structure calculations in the region of small gaps during the ML-accelerated TSH trajectory propagation^8,9,31^. To mitigate this issue, specially designed protocols, such as interpolation from minimum energy conical intersection points^14,26,27^ were sometimes employed.

**Fig. 3:**
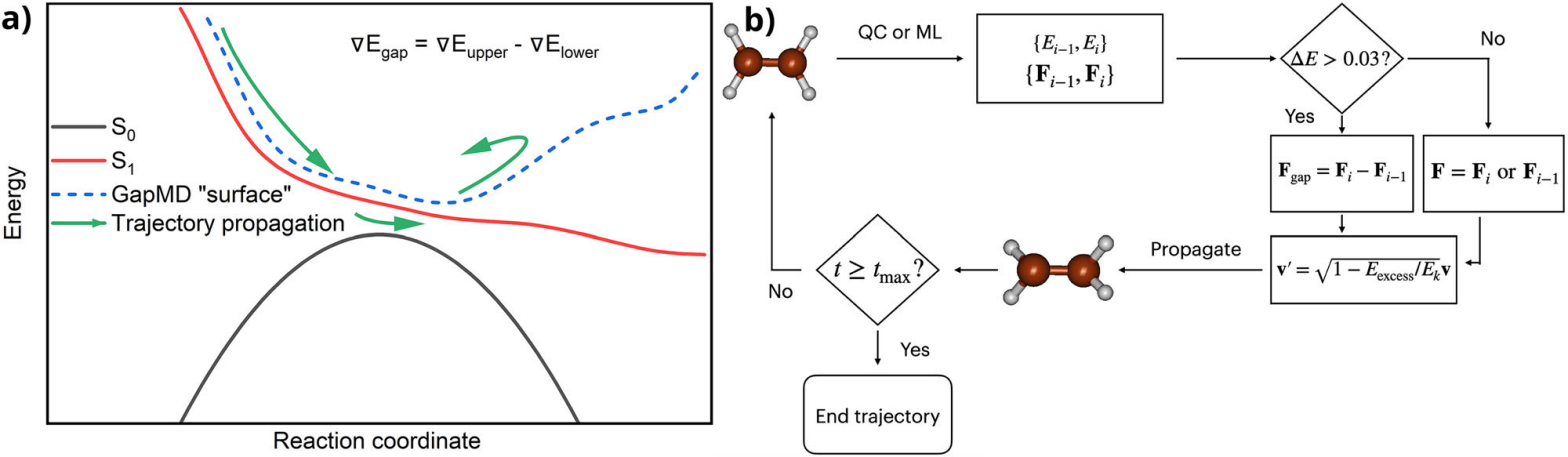
Gap dynamics. **a** Shows a schematic depiction of the gap-driven dynamics trajectory propagation, while (**b**) is an algorithmic flowchart of the propagation scheme.

Here, we have developed an accelerated sampling approach to meticulously chart the small-gap regions in the vicinity of conical intersections. We use this approach to improve the robustness and accelerate the active learning loop described in the next section, but we envision that it can also be used in other contexts, such as accelerating the search for conical intersections themselves. Our approach samples the points relevant to the TSH via propagating special, gap-driven molecular dynamics (gapMD) trajectories (Fig. 3).

The gapMD trajectories involve the back-and-forth switching of propagation along the energy gap gradient and energy gradients of the adiabatic surfaces. In the region of gaps, Δ*E*, larger than 0.03 Hartree, the trajectories are propagated along the energy gap gradient, ∇*E*_gap_, defined as the difference between energy gradients in the upper, (∇*E*_upper_), and lower, (∇*E*_lower_), surfaces:

3 $$\nabla {E}_{{\rm{gap}}}=\nabla {E}_{{\rm{upper}}}-\nabla {E}_{{\rm{lower}}},$$

with equivalent expression formulated in terms of forces:

4 $${{\bf{F}}}_{{\rm{gap}}}={{\bf{F}}}_{{\rm{upper}}}-{{\bf{F}}}_{{\rm{lower}}}.$$

These trajectories, by construction, drive the dynamics into the regions with smaller gaps. The threshold of 0.03 Hartree is chosen because it is a typical range of gaps at which the interstate hoppings commonly happen^40^. Once the region of a smaller gap is reached, we switch to propagating trajectories using the energy gradients of either the upper or lower surface (see below). This is done for several reasons. Firstly, propagating exclusively with the energy gap gradients will sample many irrelevant points, e.g., towards the complete dissociation of a molecule, as the dissociated structures have degenerate energy levels with a zero gap. Furthermore, in the actual TSH, trajectories are propagated either on the upper or lower surface, as opposed to propagating along the gradient difference, which does not correspond to any of the adiabatic PESs. To ensure sampling relevant points of the phase space, the trajectory is switched to one of the surfaces that can be populated in TSH. Hence, our trajectories propagated with the energy-gap gradients are only for biasing dynamics to visit the small-gap region faster and enrich the sampling of this region.

Switching back and forth from energy gap gradients to electronic state gradients makes the gapMD not energy-conserving. To ensure energy conservation and to avoid obtaining dissociated and otherwise nonphysical geometries, if the excess energy *E*_excess_ is smaller than the current kinetic energy *E*_*k*_ of the system, the atomic velocities **v** are scaled along the momenta, i.e., according to the formula:

5 $${{\bf{v}}}^{{\prime} }=\sqrt{1-\frac{{E}_{{\rm{excess}}}}{{E}_{k}}}{\bf{v}}.$$

The updated velocities ${{\bf{v}}}^{{\prime} }$ are used in the further propagation. The excess energy, *E*_excess_, is defined as a difference between the total energy at the current time step and the initial total energy at the time step zero. If the excess energy is larger than the kinetic energy at the current time step, it means that there is not enough kinetic energy to compensate for this excess in the system. Hence, in such a case, we switch the trajectory propagation to the electronic state surface regardless of the gap value.

### End-to-end active learning targeting accurate hopping probabilities

**Fig. 4:**
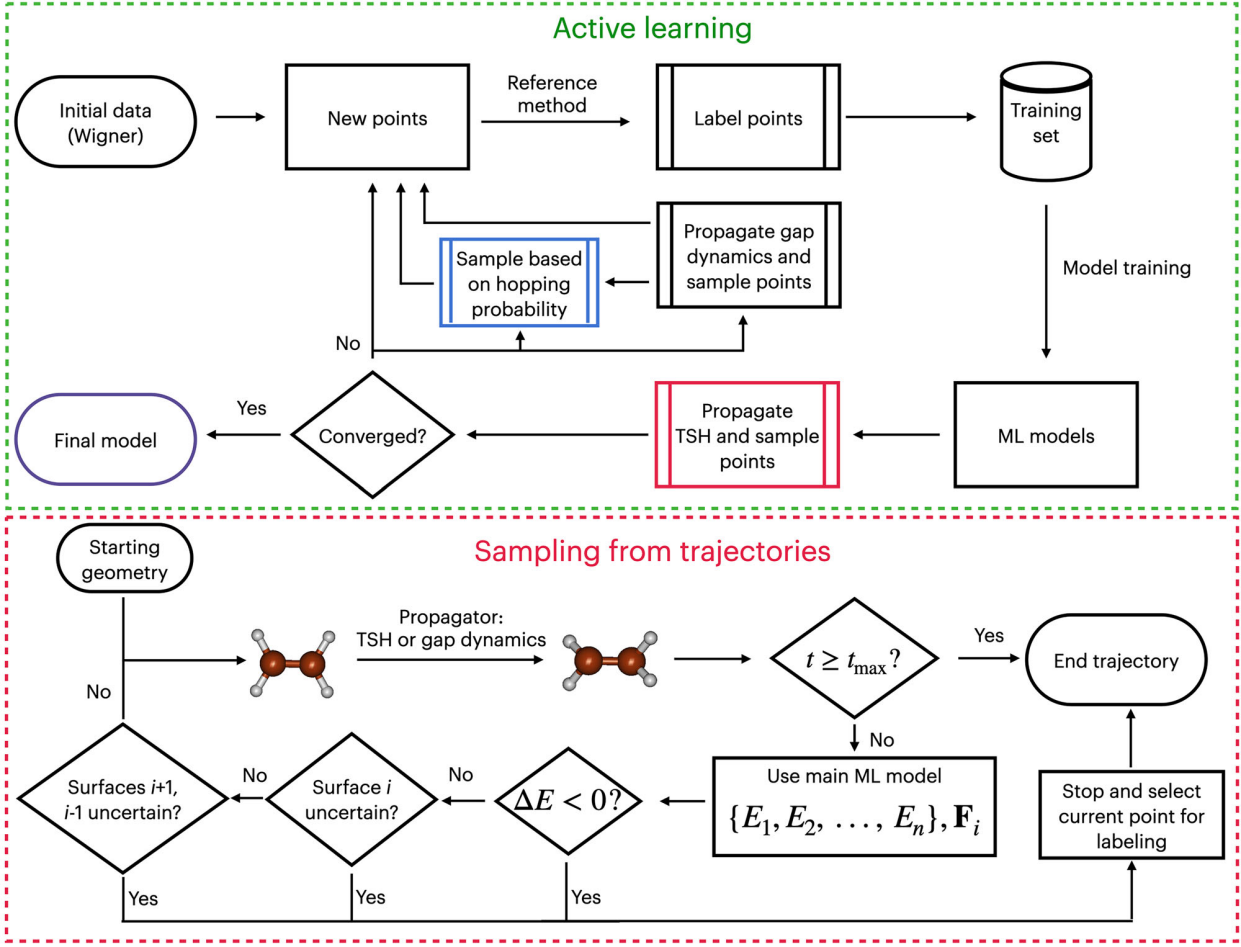
Flowchart describing the active learning procedure. The upper panel shows the full loop of the procedure, from sourcing the initial dataset, labeling with a reference method of choice, training the main and auxiliary ML models used to propagate TSH simulations, from which points are sampled, later propagation of gapMD dynamics, and finally sampling points based on hopping probability. The bottom panel details how TSH and gapMD trajectories are propagated, where the three criteria used to sample points: negative energy gaps, current surface uncertainty, and adjacent surface uncertainty.

Ultimately, our goal is to design a protocol for robust ML-TSH through meticulous charting of the electronic-state manifolds of new systems from scratch. The multi-state models and gapMD provide useful tools for achieving this goal. Hence, we incorporate both tools into the end-to-end, physics-informed active learning (AL) protocol (Fig. 4). This AL protocol ensures not only the sampling quality of the adiabatic surfaces and small-gap regions but also that the hopping probabilities are accurate.

The core of the AL protocol is running many trajectories to sample new points, labeling them (i.e., calculating the reference electronic-structure properties), training the ML model on the data updated with the newly labeled points, and repeating the procedure until converged. We used our domain knowledge to tune all its stages to obtain robust ML-TSH results as efficiently as possible.

We generate the initial data set using statistical considerations in the same way as described previously for our AL procedure for the ground-state molecular dynamics^41^. In brief, the points are sampled until the validation error does not drop too much (judged by training the ground-state energy-only model). From a ground-state minimum geometry of the studied molecule, 50 conformations are sampled from a harmonic-oscillator Wigner distribution^42^, and the error in energy prediction is calculated using a 5-fold cross-validation. The procedure is repeated until the projected accuracy improvement is less than 10%, as estimated by fitting the learning curve.

Once the initial data is sampled, we start the AL loop by training our multi-state model on both energies and energy gradients of all electronic states and propagating ML-TSH trajectories started from a selected number of Wigner-sampled initial conditions (atomic positions and velocities), which was set to 50 in the current work. At each time step, we check several criteria to evaluate whether to propagate or to stop the trajectory and sample the geometry at this time step (Fig. 4, bottom). The first criterion is whether the ML-predicted energy gap between the current and adjacent surfaces is negative: this check ensures correct state ordering. Then, we check whether the uncertainty quantification (UQ) metric for the current surface exceeds the threshold, which ensures the quality of the relevant adiabatic surfaces on which the dynamics are propagated. Finally, we check whether the UQ for the surfaces up and down on the current surface exceeds their respective thresholds. This ensures that the crucial energy gaps are monitored indirectly. It is also possible to check the UQ for the energy gaps involved directly, but our tests showed that this does not enhance the model performance, particularly when gapMD is used (see below).

The UQ values, *U*, are obtained using our previous, physics-informed scheme^41^, which was developed for ground-state dynamics. The underlying assumption of this procedure is that the shape of the potential energy surfaces must be contained in the sampled points used for training ML potential. If this is not the case, the model extrapolates from the given data, which can break down and lead to unphysical results. The uncertainty is checked by evaluating the absolute deviations in energy predictions by the main multi-state model trained on more physical information (energies and energy gradients), and the auxiliary multi-state model trained on less information (only on energies): *U* = ∣*E*^aux^ − *E*^main^∣. If the model starts to extrapolate the shape of the PES using the known energy gradients, then the deviation should grow with respect to the auxiliary model, which does not have access to the energy gradients. The main model is used to propagate the nonadiabatic dynamics simulations, while the predictions of the auxiliary model are only used to judge the quality of the predicted energies. This has been shown to produce robust active learning for ground-state dynamics^41,43^.

The UQ thresholds are calculated based on statistical considerations for each state separately, in the analogous way as before in the ground-state AL^41^. They are evaluated with the UQ values calculated for the initial validation set (10% of the initial data):

6 $${{\rm{UQ}}}_{{\rm{threshold}}}=M({\bf{U}})+3\cdot {\rm{MAD}}({\bf{U}})$$

where *M* is the median, and MAD is the median absolute deviation, with M + 3 ⋅ MAD ensuring a confidence level of 99%. While this threshold can be modified, our testing has indicated that lower values (2 MAD) do not improve the quality of the simulations, increasing only the time it takes to converge the AL procedure, while higher values lead to poor results. The UQ thresholds are calculated using the data available in the initial training set and fixed for the rest of the AL process. This procedure has the benefit that no manual, subjective setting of the thresholds is needed, in contrast to the adaptive sampling widely used in the field^11,21,22^.

None of the above sampling criteria directly addresses the key factor controlling surface hopping: the hopping probability. This factor is susceptible to any deviations in the energy gap, and having a model that yields accurate probabilities is essential for ensuring robust performance in TSH. To further refine the model in terms of hopping probability, after the propagation of each TSH trajectory, we evaluate their uncertainties. The UQ metrics are computed as absolute deviations between the hopping probabilities evaluated using the energies predicted by the main and auxiliary models at each time step before the UQ threshold is exceeded. We use the same formula as in the Landau–Zener–Belyaev–Lebedev (LZBL) formulation of TSH^44–48^:

7 $${P}_{j\to k}=\exp \left(\frac{-\pi }{2\hslash }\sqrt{\frac{{Z}_{jk}^{3}}{{\ddot{Z}}_{jk}}}\right),$$

where *Z*_*jk*_ is the energy gap between adiabatic states, j and k, and ${\ddot{Z}}_{jk}$ is the second-order time derivative of that gap. The LZBL formalism is also used for all TSH propagations in this work as implemented in MLatom^48,49^ due to its simplicity and because it does not require the evaluation of the nonadiabatic couplings. We identify all time steps in all trajectories where the main and auxiliary hopping probabilities deviate by more than 10% (if higher fidelity is needed, the threshold can be reduced). In the end, no more than 15 probability-uncertain points from both ML-TSH and gap-driven trajectories are randomly sampled per AL iteration to ensure a balanced training set.

To better sample points in the direct vicinity of the conical intersections, we spawn ML-gapMD trajectories with initial conditions (geometry and velocities) taken from a random time step of each ML-TSH trajectory (see the section on gap-driven dynamics). Initial conditions for gapMD are only selected from the TSH trajectory before the first uncertain time step (as judged by the negative gap and uncertainty quantification for surfaces). The ML-gapMD are subject to the same sampling and stopping criteria, i.e., UQ of the surface energies, negative gap checks, and hopping probability uncertainty. In each iteration of the active learning procedure, a given number of trajectories, usually equal to the number of ML-TSH trajectories, are spawned, following the gradient difference of two randomly selected adjacent potential energy surfaces. Half of these trajectories are selected to follow the upper potential energy surface in the small-gap region, and half of them follow the lower surface.

The convergence rate of AL is evaluated as the ratio of certain trajectories to the total number of ML-TSH trajectories. Trajectories are considered certain if they are propagated without exceeding the surface UQ thresholds or having negative gaps. Hopping probability UQ is not taken into account, as it is an exponential function of the energy gap, which is very sensitive to any deviations. If the convergence rate is greater than the desired value (95% in this work), the AL procedure is stopped, and the current model is considered converged.

At an AL iteration, the number of sampled points usually exceeds the number of trajectories because we select them from additional ML-gapMD trajectories and hopping probability conditions. However, all the points sampled in a given AL iteration are included in the training set. Alternatively, to speed up convergence, each AL iteration can proceed until a pre-selected number of sampled points is achieved, after which the models are re-trained.

### How efficient is the physics-informed AL?

To asses the efficiency of the physics-informed AL protocol, we test it by generating the training data and multi-state model for a fulvene molecule from scratch.

**Fig. 5:**
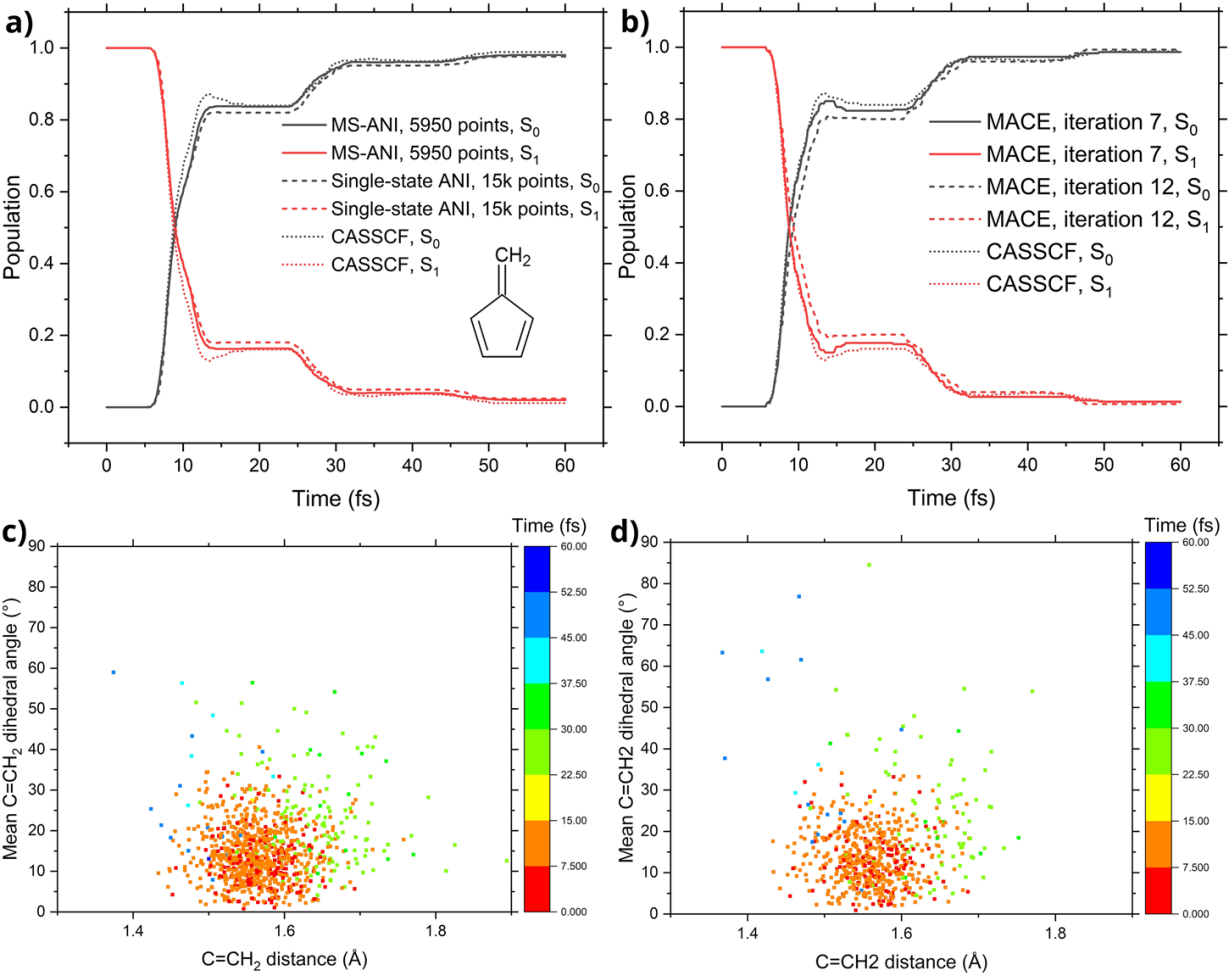
Summary of the nonadiabatic molecular dynamics simulations of fulvene. **a** Shows the electronic state population evolution during the dynamics conducted with the MS-ANI model (full lines), single-state ANI models (dashed lines), and reference CASSCF dynamics (dotted line). **b** Shows the electronic state population evolution during the dynamics conducted with the MACE models at iteration 7 (full line), iteration 12 (dashed line), and reference CASSCF populations (dotted line). **c**, **d** Show correlation plots of the C=CH2 distance and the mean dihedral angle at the S_1_ → S_0_ hopping points in ML-TSH dynamics (**c**) and reference CASSCF dynamics (**d**).

For fulvene, the AL convergence was achieved in three days on a single RTX 4090 GPU and 16 Intel Xeon Gold 6226R CPUs, only with 19 iterations: an unprecedented efficiency not reported before. The final training data set contained relatively few (5950) points. This labeling cost is equivalent to computing only ten quantum mechanical trajectories in terms of CPU time; in terms of the wall-clock time, labeling can be performed much faster as it can be efficiently parallelized. The performance of the model in ML-TSH shows excellent agreement with the reference quantum-chemical results both in terms of the populations and the distribution of the geometric parameters at the *S*_1_ → *S*_0_ hopping points (Fig. 5a, c, and d, as well as Table 1). The latter are classified into three groups, following the refs. ^50,51^ based on the C=CH_2_ bond length, and the mean dihedral angle ${\phi }_{{{\rm{C = CH}}}_{{\rm{2}}}}$ between the 5-membered ring and the methylene group:

8 $${\phi }_{{{\rm{C = CH}}}_{{\rm{2}}}}=\frac{1}{4}\left(| {\phi }_{{{\rm{C = CH}}}_{{\rm{2}}}}^{{{\rm{cis}}}_{{\rm{1}}}}| +| {\phi }_{{{\rm{C = CH}}}_{{\rm{2}}}}^{{{\rm{cis}}}_{{\rm{2}}}}| +| {\phi }_{{{\rm{C = CH}}}_{{\rm{2}}}}^{{{\rm{trans}}}_{{\rm{1}}}}| +| {\phi }_{{{\rm{C = CH}}}_{{\rm{2}}}}^{{{\rm{trans}}}_{{\rm{2}}}}| \right).$$

The planar group is defined with ${\phi }_{{{\rm{C = CH}}}_{{\rm{2}}}} < 3{0}^{\circ }$, twisted-stretched— ${\phi }_{{{\rm{C = CH}}}_{{\rm{2}}}} > 3{0}^{\circ }$ and C=CH_2_ > 1.55 Å, and twisted shrunk— ${\phi }_{{{\rm{C = CH}}}_{{\rm{2}}}} > 3{0}^{\circ }$ and C=CH_2_ < 1.55 Å.

**Table 1:** Mean value and error bars (95% confidence interval) of observables describing the deactivation channels and kinetics of fulvene for ML dynamics and reference CASSCF dynamics

Observable	CASSCF dynamics	ML dynamics
*S*_1_ Population at 20 fs (%)	16.3 ± 2.9	16.9 ± 2.3
*S*_1_ Population at 40 fs (%)	3.7 ± 1.5	3.9 ± 1.2
Planar hopping (%)	94.0 ± 1.8	93.8 ± 1.5
Twisted-stretched hopping (%)	3.7 ± 1.5	4.1 ± 1.2
Twisted-shrunk hopping (%)	2.3 ± 1.2	2.15 ± 0.90

The obtained data is also in good agreement with previous works^50,51^, reporting CASSCF dynamics of fulvene with the Baeck–An couplings.

### Do we need all these complications?

The important question is: do we truly need all these complications for high-quality ML-TSH dynamics? Indeed, we tried many different settings over eight years of research, and none showed satisfactory performance in terms of computational efficiency and robustness of the protocol. For example, a common robustness problem observed with less elaborate protocols was that the populations may deteriorate with more AL iterations. Small changes in the AL settings would sometimes lead to completely different results, too. Another issue is that, often, obtaining trajectories with reference electronic-structure method turns out less troublesome than fighting with all the instabilities of ML-TSH. As evidenced by many publications on ML-TSH, these issues can be tolerated and mitigated by experts to solve the problems that are beyond the reach of electronic-structure TSH. Still, they definitely hamper the wider adoption of the technique.

For example, when we use our physics-informed AL developed for the ground state with single-state ANI-type ML models and without gapMD and probability uncertainty evaluation, we can also obtain a good population plot for fulvene (Fig. 5a, no checks for negative gaps were performed either). The problem is, however, that the procedure converged only after a whopping 104 days, 160 iterations, and ca. 15000 training points (using NVIDIA GeForce RTX 3090 for model training and 8 CPU nodes for labeling and MD propagation). Admittedly, the populations started to look good with as few as dozens of iterations, but, in general, it should be considered risky to take such non-converged models if one does not know the reference population in advance.

One can also question whether a better ML model can remove the need for some of our special techniques. To address this, we repeated the above-simplified protocol but with the state-of-the-art, single-state MACE ML model^52^. The model is very slow, which resulted in 28 days of AL to produce 13 iterations and 3850 training points, and it eventually crashed due to memory issues. The AL was not converged either (only 77% trajectories were converged), and the last iteration’s model yielded a population that was worse than some of the previous iterations (Fig. 5, panel (b)). The major problem of the MACE model is its high cost, which might be only worth paying in special cases that we have not identified so far.

All of the above anecdotal examples illustrate that the special techniques of our final end-to-end protocol are not bells and whistles but the result of an 8-year-long struggle to obtain an efficient and robust protocol. These examples are just the tip of the iceberg of the thousands of thrown-away experiments and several generations of students and postdocs efforts.

### Does the protocol work for big systems and more states?

**Fig. 6:**
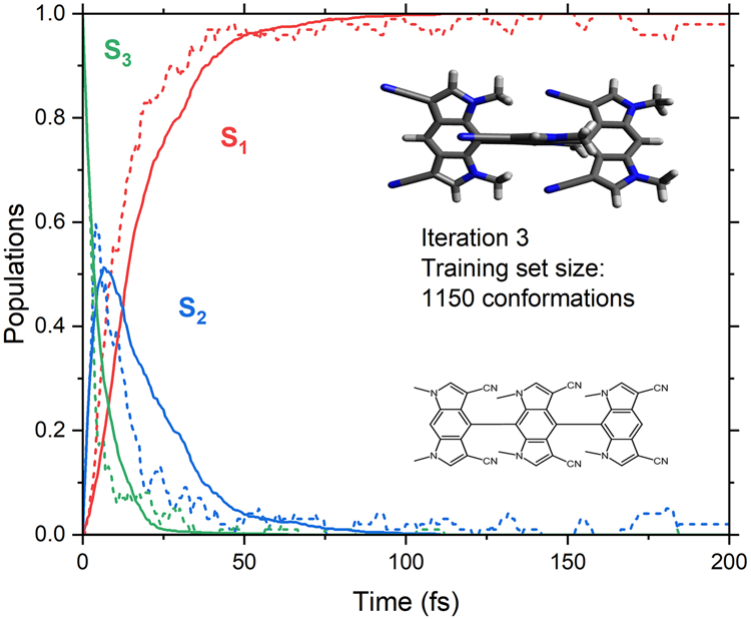
Electronic state population evolution during the dynamics of the molecular ferro-wire. Predictions of the ML model are plotted with full lines, and reference AIQM1/CIS calculations with dashed lines.

Fulvene is a simple system of small size and of only two-state-driven photodynamics. To go beyond such a simple picture, we tested our end-to-end protocol on a molecular ferro-wire made of 80 atoms and featuring four electronic states in its regular photorelaxation dynamics. The chosen molecule is characterized by a complicated electronic structure due to charge transfer between its three structural units. Amazingly, our AL procedure converged after only three iterations and produced 1150 training points. The final obtained multi-state model agrees well with the reference populations determined at the AIQM1/CIS level (Fig. 6). Testing of the model was performed using 1000 ML-TSH trajectories and compared to 100 reference AIQM1/CIS trajectories.

In this case, propagating one ML-TSH trajectory takes about 5 min on a single Intel Xeon Platinum 8268 CPU, which is a significant speed-up even with respect to fast methods such as AIQM1/CIS (having the speed of a semi-empirical method), where one trajectory takes about 1.5 CPU-hours on average. The computational efficiency of ML-TSH with MS-ANI is reported in Table S3 of the ESI, with trajectories propagated faster than 1.5 ns/day for all studied systems.

### Can the protocol learn photoreactions?

Next, we would like to see whether the proposed end-to-end protocol works for a more complicated case of photochemical transformation. For this, we take as a test case the well-known photoisomerization of azobenzene^53–55^, starting from both *cis* and *trans* isomers.

To do this efficiently, we introduce a slight modification to the AL procedure in this case. Namely, at each iteration, an equal number of trajectories is started from both the *cis* and *trans* isomers of azobenzene. This ensures that we sample both the product and the starting material of each photoprocess, greatly accelerating the model-training convergence (as is known^41^ from the AL-based search of conformers in their ground state). After running for about 3 weeks of wall-clock time, with 108 iterations (about 18000 training points), the model was 92% converged for the set of trajectories with initial conditions filtered to an excitation window of 2.53 ± 0.3 eV, corresponding to the *S*_0_ → *S*_1_ transition energy at the ground-state minimum, computed at the AIQM1 level of theory. To speed up the calculations, a broadening procedure using Wigner sampling was performed, which consisted of computing frequencies at each point of the training set, using the ML model, and using these frequencies to sample an additional point, effectively doubling the training set size. This increased the convergence to 97% and resulted in a noticeable improvement in the predicted populations. The constructed model was 98% converged for the reverse, *cis* → *trans* photoisomerization process. The final training set consisted of 35071 points. Eventually, 500 trajectories were propagated for both isomers, with the results discussed below.

**Fig. 7:**
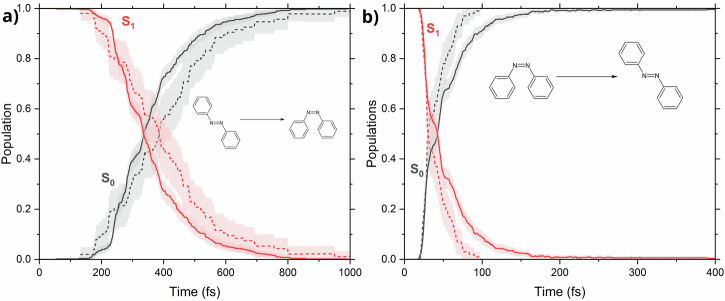
Electronic state population evolution during the isomerization dynamics of azobenzene. Trajectories shown in (**a**) started from the *trans* isomer, while trajectories starting from the *cis* isomer are shown in (**b**). ML-TSH is represented by full lines, while reference AIQM1/MRCI-SD trajectories are dashed. The shaded areas show the statistical uncertainty of the trajectories with a 95% confidence interval.

Analyzing the electronic state populations during the dynamics (depicted in Fig. 7), we see outstanding agreement between the ML model and reference AIQM1/MRCI-SD trajectories in the *trans* → *cis* isomerization reaction, as well as good agreement for the *cis* → *trans* photoprocess.

The excited-state lifetimes can be extracted by fitting an exponential function to the ground-state population rise with $P(t)=A(1-\exp (-t/\tau ))$, where *τ* is the photoprocess timescale, and *A* is associated with the infinite-time population limit. After fitting for the *trans* → *cis* isomerization, we obtained 387 ± 15 fs for the ML-TSH dynamics and 437 ± 16 fs for reference AIQM1/MRCI-SD, which are in excellent agreement. For the *cis* → *trans* process, the fitted timescale is 53.0 ± 0.8 fs for ML-TSH dynamics and 41 ± 1 fs for reference dynamics. While the predicted decay rates agree acceptably well between the ML model and reference dynamics, overall, the AIQM1 method seems to overestimate the speed of the *trans* → *cis* isomerization process, as literature data^53^ report a timescale of 74 fs at the CASSCF level.

**Fig. 8:**
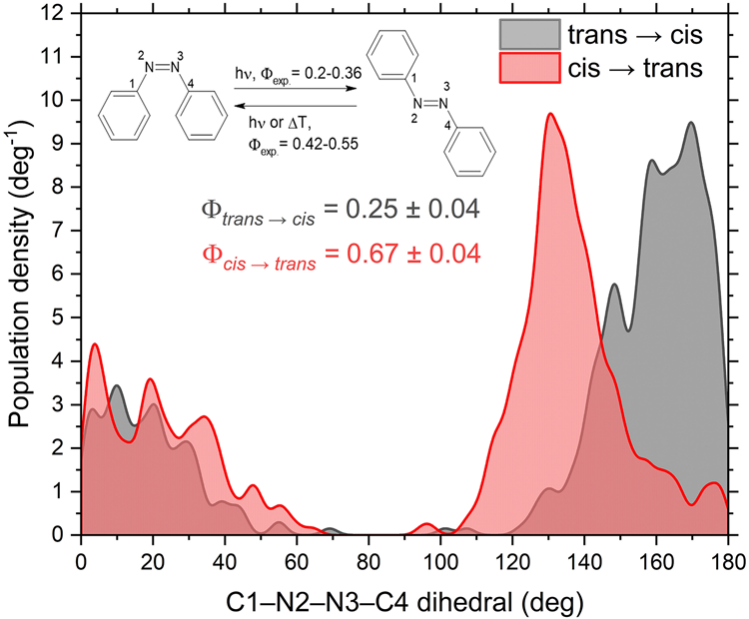
Distribution of the C–N–N–C dihedral angle of azobenzene at the trajectories final points. Distribution derived from trajectories starting from the *trans* isomer of azobenzene is marked in gray, while the distribution derived from geometries starting from the *cis* isomer of azobenzene is marked in red.

As the next point in the analysis, we can look at the predicted quantum yields of the photoisomerization processes. The dihedral angle C_1_–N_2_–N_3_–C_4_ (as shown in Fig. 8) can be used as a convenient descriptor of the formed product, taking the value of ~0° for *cis*-azobenzene, and 180° for *trans*-azobenzene. The distribution of the final dihedral angles can be found in Fig. 8, in the form of a density distribution plot. Taking a dihedral angle of fewer than 60° as a fingerprint of the *cis* isomer, and attributing the rest of the population to the *trans* isomer (no other photoproducts were observed), the simulated quantum yields *Φ* are calculated as the fraction of the trajectories relaxing to form the photoproduct (the other isomer), *N*_reactive_, to the total number of trajectories *N*_traj_: *Φ* = *N*_reactive_/*N*_traj_.

Here, the model shows excellent agreement with results obtained by the reference method, as well as the experimental results^56,57^. The ML-TSH dynamics predicts the quantum yields of ${\Phi }_{trans\to cis}^{{\rm{ML}}}=0.25\pm 0.04$ and ${\Phi }_{cis\to trans}^{{\rm{ML}}}=0.67\pm 0.04$. The uncertainties of the quantum yields were estimated using the normal approximation interval for a binomial process, assuming a confidence interval of 95%. These values agree with the predictions of the reference method, AIQM1/MRCI-SD (0.24 ± 0.09 for *trans* → *cis*, and 0.78 ± 0.10 for *cis* → *trans*), as well as with experimental results, predicting a quantum yield of 0.20–0.36 for the *trans* to *cis* isomerization. The remarkable accuracy at which the proposed model reproduces quantum yields simulated using reference methods can be attributed to the inclusion of the gap-driven trajectories in the sampling process.

### Discovering oscillations in *cis*-azobenzene photoreaction

**Fig. 9:**
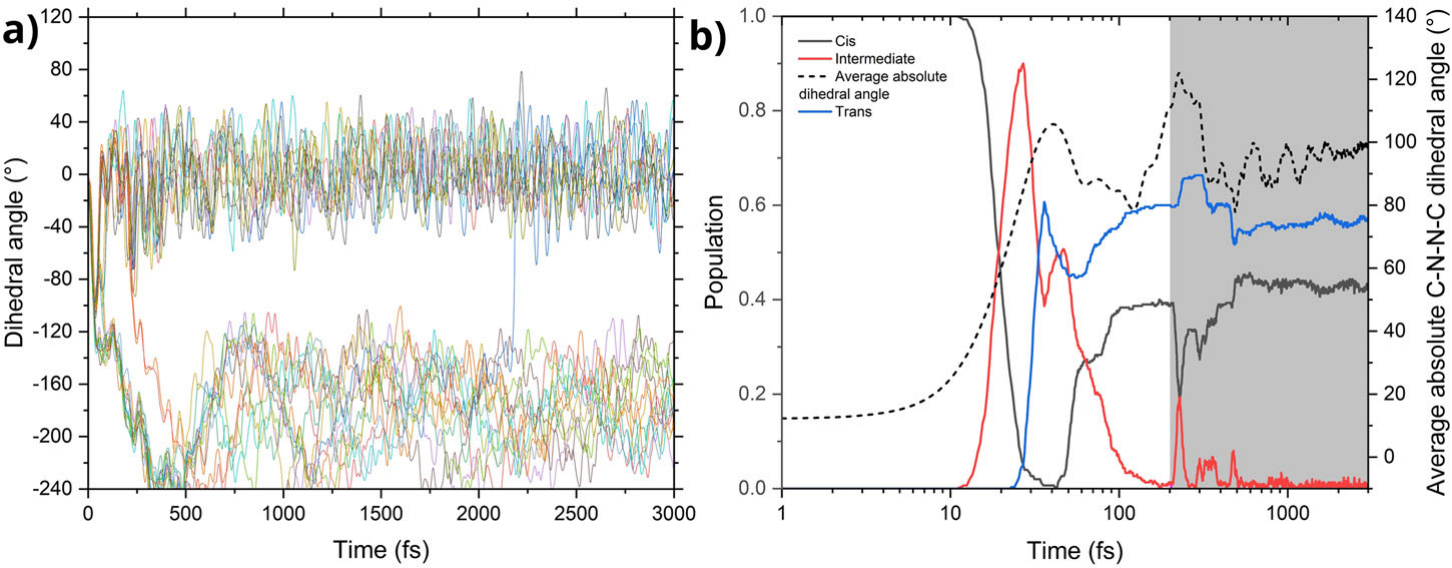
Extended dynamics of *cis-azobenzene.* **a** Evolution of the C–N–N–C dihedral angle during the dynamics, in a random subset of 25 trajectories; **b** temporal evolution of the populations of three isomers of azobenzene: *cis*-azobenzene, C–N–N–C dihedral angle of below 40°; intermediate form, dihedral angle between 40 and 140°; *trans*-azobenzene, dihedral angle above 140°; the dotted line shows the average absolute dihedral angle during the dynamics, and the shaded region of the graph indicates the time after which all trajectories decayed to the ground state. Note the logarithmic scale of the X axis.

We use the ML-ANI model trained for azobenzene to extend the timescale of the ML-TSH simulations, propagating 300 additional trajectories to 3 ps. These simulations revealed that the *cis*-*trans* photoisomerization has some features not previously reported. We found out that after relaxation to the ground state and isomerization to the *trans* isomer, the molecule is so hot that it often undergoes the reverse process of isomerization to the *cis* isomer (Fig. 9a). This back-and-forth *cis*-*trans* isomerizations have an oscillatory behavior persistent till the end of trajectory propagation, even after 100% of the trajectories decay to the ground state (Fig. 9b). This long-scale thermally induced isomerization process after the decay to the hot-ground state has not been observed in the previous, smaller scale (shorter and with fewer trajectories) NAMD studies^53,54^. These oscillations occur in a much longer time scale than those reported by Weingart et al.^58^ (which we could also reproduce with our model).

Machine learning proves to be a convenient tool in this analysis, as it allows us to rapidly compute long trajectories, which are necessary to make this observation. Additionally, it allows us to propagate arbitrarily many trajectories, making this analysis more precise. Propagating a single *cis*-azobenzene trajectory for 3 ps takes about 30 min of CPU time, compared to an estimate of 2 days using reference methods. These oscillations are not just curious observations, they have an impact on the overall quantum yield of the reaction. If one were to cut the simulations short and measure the quantum yield at around 250 fs, right after the deactivation to the ground state, the obtained value would be about 0.65. However, the final quantum yield after equilibration is noticeably different (0.56), which closely matches experimental results (typically reporting 0.42–0.55), which means that the inclusion of longer timescale thermal equilibration is necessary to correctly describe this process.

### Can the multi-state model work across different systems?

**Fig. 10:**
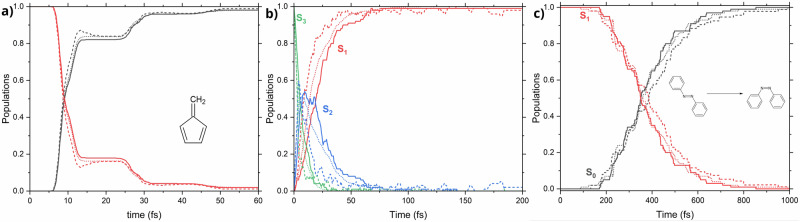
Electronic state population evolution during the dynamics conducted with a combined model. **a** fulvene, **b** molecular ferro-wire, **c** *trans* → *cis* dynamics of azobenzene. Full lines represent the populations from ML-TSH dynamics performed with the model trained on a combined training set, while dashed lines are reference populations and dotted lines are populations taken from simulations with dedicated ML models.

Since, by construction, our multi-state model must be able to learn systems of arbitrary composition, we trained it on a data set combining the AL-produced data sets of fulvene, azobenzene, and the molecular ferro-wire. These data sets were labeled with different electronic-structure methods, CASSCF, AIQM1/MRCI-SD, and AIQM1/CIS, respectively. They also had different numbers of electronic states (two in fulvene and azobenzene, and four in the ferro-wire). Regardless of these differences, the model was able to learn from this new data set, and the populations produced with this single multi-state model for each of the molecules are as good as the populations produced with the separate, dedicated multi-state models, in the cases of fulvene and azobenzene, with small deviation observed in the case of the ferro-wire (Fig. 10). This proves that a single MS-ANI model can learn the underlying photochemistry of vastly different systems, which we utilized recently for constructing a universal ML potential for excited states OMNI-P2x,^66^ which is based on an extension of the MS-ANI architecture with the all-in-one learning^67^ of different electronic-structure levels.

## Discussion

Using ML methods for simulating nonadiabatic molecular dynamics is a formidable challenge due to the necessity to train ML potentials for several electronic states across a wide range of geometries, as well as due to the intrinsic complexity of photoprocesses, requiring accurate predictions in many different regions of the PES. Owing to this, state-of-the-art ML-accelerated protocols for TSH still require extensive human expertise, as well as extreme computational resources.

Herein, we present an end-to-end protocol for active learning of TSH dynamics that requires minimal user input, delivering results of unprecedented quality for different photochemical and photophysical processes. The heart of this protocol is a new MS-ANI model that is able to make predictions for any number of excited states with unprecedented quality. We leverage the idea of physics-informed active learning combined with accelerated sampling using gap-driven trajectories to explore the vicinity of the conical intersections and sampling based on hopping probability. The thresholds for uncertainty quantification in the AL procedure are based on rigorous statistical considerations that minimize arbitrary user decisions.

This all yields a robust protocol that is able to conduct nonadiabatic dynamics investigations of photophysical and photochemical processes using affordable time and resources. The protocol has enabled a larger scale exploration of the photoisomerization of *cis*-azobenzene that uncovered an oscillatory behavior of this reaction that affects the final quantum yields. Hence, we show that such explorations require longer timescale propagations which are now doable with ML.

Finally, the presented models can simultaneously predict accurate dynamics across very different chemical species with no noticeable decrease in accuracy, making them extendable across chemical space. It remains to be seen how multi-state learning performs across different systems which are more similar in terms of their structure and excited state energies. Encouragingly, multi-state learning has been recently successfully applied to learn across diverse molecular systems leading to the universal OMNI-P2x potential for excited states.^66^

## Methods

All computations were performed with the development version of MLatom^48,49^ and additional scripts, which are available in MLatom version 3.10+. Wigner sampling was performed with MLatom routines adapted^48^ from Newton-X^42^. The bulk of the computations and implementations reported here were performed on the cloud computing service of the Xiamen Atomistic Computing Suite at http://XACScloud.com that supports collaborative work. MS-ANI and ANI models are based on MLatom’s interface to the TorchANI^59^ package. AIQM1 calculations were performed with the help of MLatom’s interfaces to the MNDO program^60^ (providing the semi-empirical part^61^ of AIQM1), TorchANI (providing ANI-type NN), and dftd4^62^ programs. CASSCF calculations were performed through the interface to the COLUMBUS quantum chemistry package^63^.

### Fulvene

For the active learning of fulvene, initial points were sampled from a harmonic approximation Wigner distribution, with a total of 250 points. The maximum propagation time was set to 60 fs with a time step of 0.1 fs. Velocities after hopping were rescaled in the direction of the momentum vector, using a reduced kinetic energy reservoir^51^. In each iteration of the AL procedure, 50 ML-TSH trajectories were run, with an additional 50 ML-gap-driven trajectories (25 on each electronic surface). From this set, 15 additional points were sampled based on hopping probability uncertainty. Points were sampled in this manner until a threshold of 300 points was reached in each iteration, after which the new models were trained.

Reference calculations were performed using the CASSCF method, with an active space of 6 electrons in 6 orbitals. The same settings were used for AL loops with the single-state ANI model and the MACE model. However, these runs did not include accelerated sampling with gap-driven trajectories or sampling based on hopping probability.

Testing of the model was performed using 1000 trajectories for the MS-ANI model, as well as the single-state ANI models, and 300 trajectories for the MACE models (due to extensive computational costs). 623 CASSCF(6,6) trajectories were used as reference.

### Ferro-wire

In the active learning procedure of the ferro-wire, initial points were sampled from a Wigner distribution using the standard procedure, with a total of 250 points. The maximum propagation time was set to 200 fs with a time step of 0.5 fs. The velocities after hopping were rescaled in the direction of the momentum vector using a reduced kinetic energy reservoir. Points were sampled using the same procedure as for fulvene. Reference calculations were performed using the AIQM1/CIS method with default settings. All trajectories were initialized in the third excited state, *S*_3_, without filtering. 100 ML-TSH trajectories were propagated with the model trained on the combined fulvene/ferro-wire dataset.

### Azobenzene

In the active learning procedure of azobenzene, initial points were sampled from the Wigner distribution using the standard procedure, with a total of 250 points. The maximum propagation time was set to 1000 fs for trajectories starting from the *trans* isomer and 400 fs for *cis*-azobenzene, with a time step of 0.5 fs. The velocities after hopping were rescaled in the direction of the momentum vector using a reduced kinetic energy reservoir. In each iteration, two sets of trajectories were propagated, one starting from the *cis* isomer and the other starting from the *trans* isomer. Model training was performed after sampling all points resulting from these trajectories, as well as a set of 50 gap-driven trajectories (25 on each active surface), with 15 points added from probability-based sampling without a maximum number of sampled points. In the active learning procedure, all trajectories were initialized in the first excited state without filtering.

Reference calculations were performed using the AIQM1/MRCI-SD method^64^. In the initial, semi-empirical part of AIQM1, the half-electron restricted open-shell Hartree-Fock formalism^65^ was used in the SCF step, with the HOMO and LUMO orbitals singly-occupied. Two additional closed-shell references were added in the MRCI procedure, a HOMO–HOMO configuration and a doubly excited, LUMO–LUMO configuration. The active space consisted of 8 electrons in 10 orbitals (four occupied orbitals, six unoccupied). Single and double excitations within a such-defined active space were allowed. 88 trajectories starting from the *trans* isomer, and 92 starting from *cis*-azobenzene were used as reference.

For Wigner sampling of initial conditions, all frequencies smaller than 100 cm^−1^ were set to 100 cm^−1^ to avoid sampling unphysical structures^48^. Testing of the model was performed on a set of initial conditions filtered to an excitation window of 2.53 ± 0.3 eV for *trans*-azobenzene and 2.89 ± 0.3 eV for *cis*-azobenzene, which corresponds to the *S*_0_ → *S*_1_ transition energies at the AIQM1 optimized ground-state minima.

In the 3 ps simulations of azobenzene, 304 trajectories were propagated, with 4 removed from the analysis set due to reaching unphysical, highly distorted geometries, leaving a total of 300 trajectories. The source of this error is most likely extending the timescale of the dynamics beyond the timescale of the AL procedure. Nevertheless, as the fraction of trajectories exhibiting this problem is very small (ca. 1%), we believe it has no impact on the end result. Then, at each point of simulations, the conformation of the molecule was classified into either: *cis*-azobenzene, for a C–N–N–C dihedral angle of below 40°, an intermediate form with the dihedral angle between 40 and 140°, and *trans*-azobenzene, with the dihedral angle above 140°. The populations of these three forms are then averaged out over all of the trajectories.

## Supplementary information

Supporting Material for

## Data Availability

The data (training sets and ML models) is available under the open-source MIT license at https://github.com/dralgroup/al-namd.

## Code availability

The code is available in the open-source MLatom under the MIT license as described at https://github.com/dralgroup/mlatom.
