# Supplementary material for: Charting electronic-state manifolds across molecules with multi-state learning and gap-driven dynamics via efficient and robust active learning
Source: NPJ Comput Mater. 2025 May 13;11(1):132. doi: 10.1038/s41524-025-01636-z (PMC12074996; doi:10.1038/s41524-025-01636-z)
Supplement: Supplementary file 1 — Supporting Material for [file 41524_2025_1636_MOESM1_ESM.pdf]

Supporting Information for "Charting electronic-state manifolds across molecules with multi-state learning and gap-driven dynamics via efficient and robust active learning"

Mikolaj Martyka[1], Lina Zhang[2], Fuchun Ge[2], Yi-Fan Hou[2], Joanna Jankowska[⋆,1], Mario Barbatti[⋆,3,4], and Pavlo O. Dral[⋆,2,5]

[1]Faculty of Chemistry, University of Warsaw, Pasteura 1, Warsaw, 02-093, Poland
[2]State Key Laboratory of Physical Chemistry of Solid Surfaces, College of Chemistry and Chemical Engineering, and Fujian Provincial Key Laboratory of Theoretical and Computational Chemistry, Xiamen University, Xiamen, Fujian 361005, China
[3]Aix Marseille University, CNRS, ICR, Marseille, France
[4]Institut Universitaire de France, 75231 Paris, France
[5]Institute of Physics, Faculty of Physics, Astronomy, and Informatics, Nicolaus Copernicus University in Toruń, ul. Grudziadzka 5, 87-100 Toruń, Poland

Contents

S1 Performance of MS-ANI **S2**

S2 Uncertainty quantification thresholds **S3**

S3 Computational performance **S3**

S1 Performance of MS-ANI

Table S1: Performance of the MS-ANI and single-state ANI models for predicting the energies of the first eight electronic states of pyrene, kcal/mol.

State	Single-state RMSE	Multi-state RMSE	Single-state MAE	Multi-state MAE
S_0	1.32538	0.96832	1.01809	0.74647
S_1	1.33954	1.00046	1.04498	0.77016
S_2	1.29239	1.02189	1.01633	0.80134
S_3	2.1493	1.47939	1.6647	1.15242
S_4	1.92178	1.49865	1.48902	1.18519
S_5	1.72372	1.23457	1.35766	0.98109
S_6	1.96994	1.28371	1.52209	1.0147
S_7	1.64697	1.33066	1.28604	1.05033

Table S2: Performance of the MS-ANI and single-state ANI models for predicting the energy gaps of the first eight electronic states of pyrene, kcal/mol.

States	Single-state RMSE	Multi-state RMSE	Single-state MAE	Multi-state MAE
S_0 - S_1	1.72368	1.32452	1.30568	1.02068
S_1 - S_2	1.54319	1.23684	1.1957	0.94517
S_2 - S_3	2.38496	1.89723	1.83622	1.47973
S_3 - S_4	2.73568	2.09397	2.13051	1.68351
S_4 - S_5	2.47677	1.93942	1.91491	1.54836
S_5 - S_6	2.19074	1.42764	1.69054	1.15271
S_6 - S_7	2.1447	1.22756	1.63729	0.97736

Table S3: Difference between the MAE of single-state ANI models and MS-ANI in terms of energies and energy gaps.

Quantity	S_0	S_1	S_2	S_3	S_4	S_5	S_6	S_7
Energy MAE	0.27161	0.27482	0.21499	0.51228	0.30383	0.37657	0.50739	0.2357
Gap MAE	-	0.28500	0.25054	0.35649	0.44701	0.36655	0.53783	0.65992

S2 Uncertainty quantification thresholds

Table S4: Uncertainty quantification thresholds for AL runs described in the main text, in kcal/mol.

System	S_0	S_1	S_2	S_3
Fulvene, MS-ANI	5.58	7.72	-	-
Fulvene, MACE	4.06	9.75	-	-
Ferro-wire	27.02	24.46	21.23	34.79
Azobenzene	11.06	8.75	-	-

S3 Computational performance

Table S5: Computational performance of MS-ANI for the three studied systems, in ps/day.

System	Fulvene	Ferro-wire	Azobenzene
Computational performance [ps/day]	2725	1660	2340
